# Characterization and *Ex Vivo* evaluation of an extracorporeal high‐intensity focused ultrasound (HIFU) system

**DOI:** 10.1002/acm2.13074

**Published:** 2021-08-04

**Authors:** Yufeng Zhou, Bryan W. Cunitz, Barbrina Dunmire, Yak‐Nam Wang, Steven G. Karl, Cinderella Warren, Stuart Mitchell, Joo Ha Hwang

**Affiliations:** ^1^ School of Mechanical Engineering Northwestern Ploytechnical University Xi'an China; ^2^ Division of Gastroenterology Department of Medicine University of Washington Seattle WA USA; ^3^ Center for Industrial and Medical Ultrasound Applied Physics Laboratory University of Washington Seattle WA USA

**Keywords:** acoustic nonlinearity, characterization, *In‐situ* acoustic energy, high‐intensity focused ultrasound, lesion

## Abstract

**Background:**

High‐intensity focused ultrasound (HIFU) has been in clinical use for a variety of solid tumors and cancers. Accurate and reliable calibration is in a great need for clinical applications. An extracorporeal clinical HIFU system applied for the investigational device exemption (IDE) to the Food and Drug Administration (FDA) so that evaluation of its characteristics, performance, and safety was required.

**Methods:**

The acoustic pressure and power output was characterized by a fiber optic probe and a radiation force balance, respectively, with the electrical power up to 2000 W. An *in situ* acoustic energy was established as the clinical protocol at the electrical power up to 500 W. Temperature elevation inside the tissue sample was measured by a thermocouple array. Generated lesion volume at different *in situ* acoustic energies and pathological examination of the lesions was evaluated *ex vivo*.

**Results:**

Acoustic pressure mapping showed the insignificant presence of side/grating lobes and pre‐ or post‐focal peaks (≤−12 dB). Although distorted acoustic pressure waveform was found in the free field, the nonlinearity was reduced significantly after the beam propagating through tissue samples (i.e., the second harmonic of −11.8 dB at 500 W). Temperature elevation was <10°C at a distance of 10 mm away from a 20‐mm target, which suggests the well‐controlled HIFU energy deposition and no damage to the surrounding tissue. An acoustic energy in the range of 750–1250 J resulted in discrete lesions with an interval space of 5 mm between the treatment spots. Histology confirmed that the lesions represented a region of permanently damaged cells by heat fixation, without causing cell lysis by either cavitation or boiling.

**Conclusions:**

Our characterization and *ex vivo* evaluation protocol met the IDE requirement. The *in‐situ* acoustic energy model will be used in clinical trials to deliver almost consistent energy to the various targets.

## 1 INTRODUCTION

Ultrasound is an effective, low‐cost, and non‐ionizing diagnostic modality used for several decades in clinics. Recent years have seen a dramatic interest in its application as a surgical and therapeutic tool, especially high‐intensity focused ultrasound (HIFU) for tissue ablation in the treatment of cancers and solid tumors.^1^ High‐intensity focused ultrasound energy can be focused inside the human body to raise the local temperature above 65°C within seconds.^2^ Since the mid‐1990s, advancements in clinical instrumentation have brought HIFU from the lab to the medical mainstream. In China and Europe, more than 100 000 patients have been involved for treatment of uterine fibroids, and cancers of the prostate, liver, kidney, breast, pancreas, brain, and bone.^2^, ^3^, ^4^ In comparison to traditional cancer treatment methods (i.e., open surgery, radiotherapy, or chemotherapy) and other physical methods for tissue ablation (i.e., lasers, microwave, or radiofrequency ablation), HIFU has advantages of being a non‐invasive and local treatment, deep penetration, better selectiveness without damaging adjacent vital structures, easier power control, and non‐ionizing radiation.^1^

International Electrotechnical Commission (IEC) has published several standards on ultrasound diagnostic, continuous‐wave or pulsed Doppler, physiotherapy system, and pressure pulse lithotripter.^5^, ^6^, ^7^, ^8^ Medical ultrasound fields are typically characterized in water by measuring the spatial and temporal distribution of pressure using a piezoelectric (PVDF membrane or needle) hydrophone and the acoustic power (up to 30 W, i.e., ultrasound power meter from Ohmic Instruments) using an air‐backed metal cone that intercepts the entire field. The acoustic intensity is derived from the measured pressure waveform assuming that the local pressure and particle velocity are in‐phase (“plane‐wave assumption”). However, the existing techniques may not be appropriate for characterizing HIFU fields due to the strong nonlinearity and potential cavitation damage to the hydrophone.^9^ A fiber optic probe hydrophone (FOPH) was used in measuring the acoustic field with high intensity and focusing gain.^10^, ^11^, ^12^ The FOPH has a small sensing element (100 μm), broad bandwidth (50 MHz or higher after de‐convolution), and a large half aperture angle of 30° and a new fiber tip can be easily prepared and self‐calibrated if cavitation damage occurs. In addition, a radiation force balance with the acoustic absorbing target placed between the source and the focus was designed for calibrating the HIFU transducers with no damage, and a minimal temperature rise was found at the electrical power up to 230 W.^13^ Until now, there is no consensus and standard on calibration and methods for describing the HIFU field [i.e., American Institute of Ultrasound in Medicine (AIUM), FDA, National Institute of Standards and Technology (NIST) guideline],^9^ except a national one in China.^14^ Therefore, accurate and reliable HIFU calibration protocols are in a great need for both product development and clinical application.

One of the most important aspects of oncology therapy is delivering an appropriate dose, which is dependent on the type and stage of cancer, radiation method, whether administered before or after surgery, and the degree of surgery success.^15^ However, the definition of dose varies with the treatment modality. In chemotherapy, the dose is determined by the patient’s body surface area and severity of the disease; radiotherapy dose is usually measured in gray (Gy), exposure per unit mass of a medium to be treated; a temporal temperature relationship is used as the thermal dose in RFA. In HIFU literature, authors commonly reported the acoustic output (intensity and power) and exposure parameters in describing the HIFU field and exposure energy.^1^ Although the term of treatment “dose” was sometimes used, its definition is not consistent with various targets (i.e., tissue type and propagation distance through biological tissue), the HIFU transducers (i.e., geometries and working frequency), and operation parameters (i.e., burst duration, duty cycle, and the total exposure time). The thermal dose of a 240‐min exposure at 43°C is required to create thermally irreversible damage in most tissue types.^16^ However, accurate *in situ* measurement of temperature profile or calculation of thermal dose is still challenging due to the high heating rate of HIFU and low temporal resolution of thermometry.^9^ Therefore, a definition of dose in HIFU therapy, the amount of acoustic energy deposited in the tissue similar to the “absorbed dose” used in x‐irradiation, is vital for guidance in HIFU application.

An extracorporeal HIFU system applied for the investigational device exemption (IDE, #100169) to the Food and Drug Administration (FDA) for clinical trials. The characterization of the produced acoustic field, the safety of HIFU ablation (i.e., temperature elevation to the surrounding tissue away from the target), estimation of the acoustic energy delivered to the target, and evaluation of *ex vivo* performance were required. In this study, the acoustic field and power were measured by a fiber optic probe hydrophone and a radiation force balance system, respectively, with the electrical power up to 2000 W. A method of delivering an *in‐situ* acoustic energy was established. Subsequently, the performance of the extracorporeal HIFU system was evaluated *ex vivo*. The relationship between the size and volume of HIFU‐generated lesions with the acoustic energy was investigated. In addition, the temperature elevation in the focal region was measured by a thermocouple array.

## 2 MATERIALS AND METHODS

### 2.A HIFU system

A clinical extracorporeal HIFU system (FEP‐BY02, Beijing Yuande Biomedical Engineering Inc., China) was used in this study. It has two identical HIFU transducers (upper and lower one for the treatment at the prone or the supine position, respectively), each consisting of 251 individual lead zirconate titanate (PZT) elements (frequency of ~1 MHz and diameter of 16 mm) driven all in phase and positioned on a spherical surface. The HIFU transducer has an outer diameter of 37 cm, an inner diameter of 12 cm, and a radius of curvature of 25.5 cm. An ultrasound imaging probe (S3, GE, Seongnam, Korea) aligned coaxially with the HIFU transducer was connected to an ultrasound imaging system (Logiq 5, GE) to identify the region of interest (ROI). The operator specified the location, size, and shape of the treatment target, electrical power to the HIFU transducer, HIFU on and off time, the number of pulses per treatment spot, and the interval spacing between treatment spots in the control software. The ROI was treated in a raster scanning pathway. Before each treatment, the water was degassed with an oxygen concentration of <4 mg/L.

### 2.B Acoustic pressure waveform and field mapping

Pressure waveforms of the lower HIFU transducer in a short‐pulse low‐power mode (100 μs pulse duration and electrical power of 17 W) were measured using a capsule hydrophone (HL0085, Onda Corp., Sunnyvale, CA) that was attached to the treatment table by a custom‐built fixture. At least five digitized pressure waveforms were obtained from an oscilloscope (9304CM, LeCroy, Chestnut Ridge, NY) at each position. The acoustic field was mapped in a resolution of 0.25 mm over a 30 mm × 30 mm region in the focal (*x‐y*) plane and a 10 mm × 85 mm region in the axial (*x‐z*) plane, respectively. Because the capsule hydrophone only works in the low‐intensity acoustic field, a FOPH (2000, RP Acoustics, Leutenbach, Germany) was used at the high output (250–2000 W). Acoustic pressure waveforms were measured at 3 different locations in the HIFU axis: *z* = −50, −8, and 0 mm, corresponding to the locations of grating‐lobes, the first prefocal side‐lobe, and focal point of the HIFU transducer, respectively. The power spectra were calculated from the measured pressure waveforms using a Welch algorithm.^17^ Search for the acoustic focal point, waveform measurement, and field scanning were controlled by a lab‐written LabView (National Instruments, Austin, TX) program.^11^

The fresh bovine livers were obtained from a local slaughterhouse (Schenk Packing, Stanwood, WA), immediately immersed in phosphate‐buffered saline (PBS) solution, and chilled on ice. Within 2 h they were cut into the size of ~20 × 20 × 6 cm (L × W × H) with the capsule left intact as the acoustic wave entry site, and attention was paid to exclude major vessels for consistent lesion production. After being degassed, the liver sample was positioned about 10 mm proximally to FOPH which was aligned to the HIFU focus in the free field. Then the focus of the HIFU beam after propagating through tissue was found using the acoustic field mapping method mentioned above. FOPH was realigned for the acoustic pressure waveform measurement with the presence of tissue samples.

### 2.C Acoustic power measurement

**Fig. 1:**
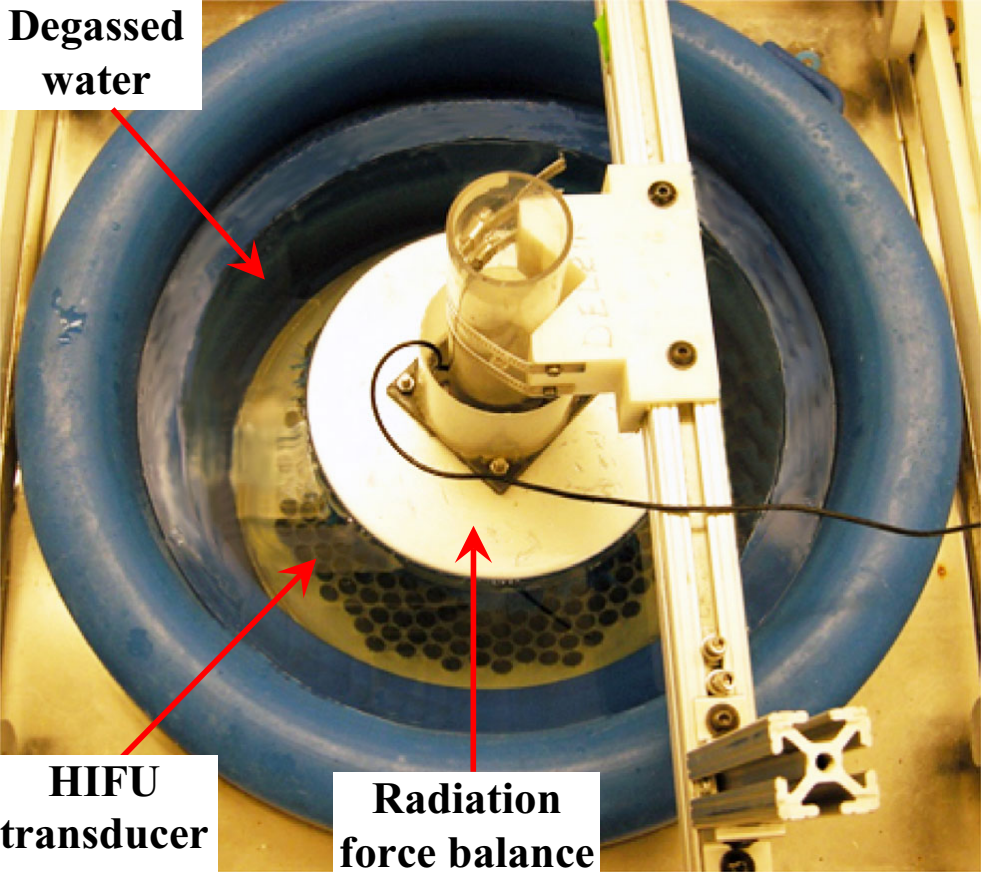
Acoustic power measurement of the lower high‐intensity focused ultrasound transducer by a lab‐built radiation force balance system.

The acoustic power output from the HIFU transducer was measured with a lab‐built radiation force balance (Fig. 1). Nylon brush bristles with a diameter of 0.44 mm and length of 3.5 cm were embedded in a silicone elastomer (220 g, Sylgard 170, Dow Corning, Midland, MI) mixed with Nickel powder (44 g, −400 mesh, Alfa Aesar, Ward Hill, MA) and plastic microspheres (PM6545, PQ Corp, Valley Forge, PA) to absorb the ultrasound energy. The absorber in a diameter of 250 mm was suspended from a load cell (SML‐100, Interface, Scottsdale, AZ) whose signal was digitized by a data acquisition (DAQ) board (SCB‐68, National Instruments) at a sampling frequency of 1000 Hz. The sensitivity of the radiation force balance was calibrated using a weight set (VWR, West Chester, PA) each time before measurement. Then the position and air being trapped on the surface of the acoustic absorber were examined in the sonography. The discrepancy of the stabilized responses between HIFU on (3 s) and off (5 s) stage was used to calculate the radiation force, *F*, and the subsequent acoustic power output, *P_A_*,^14^

(1) $$P_{A}=NFc\left(corr\right)e^{2\alpha L}/\sum_{i=1}^{n}\cos\vartheta_{i}$$

where *N* is the number of PZT elements, $\vartheta_{i}$ is the angle between the acoustic beam axis of the *i*‐th element and the main axis of the HIFU array, *α* is the absorption of the propagation media, *L* is the distance between the center of the transducer and the absorbing target (usually L≤0.7·D.), *D* is the focal length, *corr* is the planar wave correction factor of a piston element,^14^

(2) $$corr=\frac{1‐J_{1}\left(2ka\right)/ka}{1‐J_{0}^{2}\left(ka\right)‐J_{1}^{2}\left(ka\right)}$$

where *k* is the wave number, *a* is the activating sensor size of a single piston, J_n_ (*) is the *n*‐order Bessel function.^14^ The electrical power to the HIFU transducer was determined by

(3) $$P_{E}=V_{\mathrm{rms}}^{2}G$$

where *V_rms_* is the RMS voltage to the HIFU transducer measured by a high‐voltage probe (PPE‐2kV, LeCroy), *G* is the conductance of the HIFU transducer measured to be 17.56 ms using an impedance analyzer (4192A, Hewlett‐Packard, Palo Alto, CA). Therefore, the electrical‐to‐acoustic energy conversion ratio was calculated as follows:

(4) $$\varepsilon_{\mathrm{ff}}=\frac{P_{A}}{P_{E}}\times 100\%$$

### 2.D *Ex vivo* lesion production

The attenuation of tissue was determined using an “acoustic caliper.” A pair of transducers was mounted on a digital caliper for precise measurement of the transmission path length. A chirped pulse containing frequencies ranging from 1 to 10 MHz was transmitted first through the tissue and then through a water path (reference signal); the received tissue and reference signals were acquired and compared in order to calculate sound speed and attenuation.^18^ Measurements were repeated three times for each tissue sample. The electrical power achieving a desired and reproducible *in‐situ* acoustic energy, *E_A_*, was calculated to be

(5) $$P_{E}=\frac{E_{A}}{\varepsilon_{\mathrm{ff}}\cdot 10^{\frac{‐\alpha_{T}}{10}}\cdot \left[T_{1}\cdot \frac{n_{p}}{1000}\right]}$$

where *α_T_* is the whole attenuation through the propagation path in dB, *T_1_* is the HIFU pulse duration in ms, *n_P_* is the number of pulses per treatment spot.^19^ The electrical power was set no higher than 500 W according to the clinical experience for the high safety.

Sections of the bovine liver in the size of 4.5 × 4.5 × 6.0 cm (L × W × H) were prepared using the method described above and then transferred to a tissue‐mimicking phantom that contains 6.5% Alginate impression material (Jeltrate, Dentsply International, York, PA), taking care not to reintroduce gas.^20^ Then the center of the sample was aligned to the HIFU focus under the guidance of sonography. The relationship of HIFU‐generated lesion size and *in‐situ* acoustic energy within the range of 500–2000 J was investigated with the interval distance between the treatment spots of 7 mm, which results in a single lesion production according to preliminary observation. Furthermore, lesion interaction effects were studied by reducing the interval distance to 3–5 mm.

Immediately following HIFU ablation, the liver samples were placed in a custom‐built tissue cassette with proper maintenance of orientation and no disruption on the tissue. The samples were then frozen at −10°C overnight, ensuring sufficient stiffness to facilitate slicing while minimizing the amount of tissue expansion.^21^ Afterward, the samples were cut to record the lesion photographically in a step size of ~1 mm until no lesions were observed for at least 3 consecutive slices. The image files were then processed by a Matlab (MathWorks, Natick, MA) program. Each lesion area was fitted with an ellipse so that the area, centroid coordinates, major axis, and minor axis could be determined, from which the lesion volume was calculated as follows:

(6) $$V_{L}=\sum_{i=1}^{N}a_{i}\cdot \left(z_{i}‐z_{i‐1}\right)$$

where *N* is the number of image files, *a_i_* is the cross‐sectional area of the lesion in the *i*‐th image, *z_i_* is the incremental distance of the *i*‐th image taken in the tissue sample.

### 2.E Histological evaluation

A separate set of six bovine liver samples was treated for histological analysis. Immediately after treatment, the liver sample was carefully embedded in an optimum cutting temperature medium (O.C.T., Sakura Finetek USA, Torrance, CA), and then frozen completely on dry ice. Enzyme histochemical analysis was performed to determine cell viability with nicotinamide adenine dinucleotide‐diaphorase (NADH‐diaphorase) using similar methods to those established.^22^ Briefly, 6–8 µm unfixed frozen sections were cut and 100 µl of incubation medium (1 ml of 2.5 mg/ml α‐NADH, 2.5 ml of 2 mg/ml nitroblue tetrazolium chloride, 1 ml of 2 mg/ml PBS, and 0.5 ml Ringer’s solution, Sigma, St. Louis, MO) was placed on each section for 30 min under aerobic conditions at room temperature. After incubation, the slides were washed in distilled water for 2 min and then evaluated within 24 h of staining. Normal untreated liver and tissue sections heated to 100°C were used as negative and positive controls, respectively. Furthermore, sequential sections were also fixed in 95% EtOH, stained with H&E, and visualized on an inverted light microscope (80i Eclipse, Nikon, Melville, NY) for morphological analysis.

### 2.F Temperature measurement

**Fig. 2:**
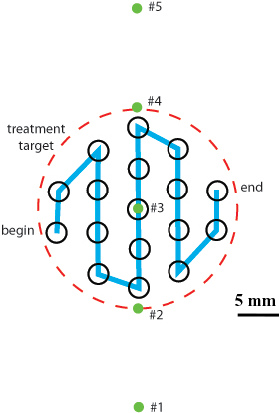
Location of thermocouples relative to the high‐intensity focused ultrasound (HIFU) target. There are a total of 17 treatment spots inside the target in a diameter of 20 mm and an interval spacing of 4 mm. The sixth thermocouple is 5 mm distal to the third one that is aligned to the HIFU focus under the guidance of ultrasound imaging.

The temperature elevation and distribution in the focal region were measured by an array of 6 type T thermocouples in a diameter of 0.3 mm (Omega Engineering, Stamford, CT). 5 thermocouples (#1–#5) were aligned in a line with 10 mm away from each other, and the sixth thermocouple was 5 mm distal to the third one that was aligned to the HIFU focus under the guidance of sonography as required by FDA for the IDE of this system (Fig. 2). To ensure a straight insertion into the tissue, the thermocouple was mounted inside a 22‐gauge needle and extended approximately 5 mm from the end of the needle. The needle was then secured into an acrylic holder which ensures the insertion depth into the tissue was the same across experiments. The treatment target is 20 mm in diameter with the interval spacing of 4 mm between spots. Thermocouple data were acquired through the SCB‐68 DAQ board concurrently with HIFU treatment. The corresponding thermal dose was calculated using the measured temperatures, *T(t)*,

(7) $$TD_{43^{\circ }C}\left(t\right)=\int_{0}^{t}R^{43‐T(\tau )}d\tau$$

with *R* = 0.25 if *T(t) <* 43°C and 0.5 otherwise.^23^ A 240‐min exposure at 43°C (240 CEM) could create thermally irreversible damage in most tissue types.^23^, ^24^, ^25^ After the ablation, the tissue was sliced into approximately 3 mm‐thick pieces to identify the presence of lesions.

### 2.G Statistics

At each experimental condition, at least six data were collected. SigmaPlot (Systat, San Jose, CA) was used to calculate the average and standard deviation and determine the data regression.

## 3 RESULTS

### 3.A Acoustic field mapping

**Fig. 3:**
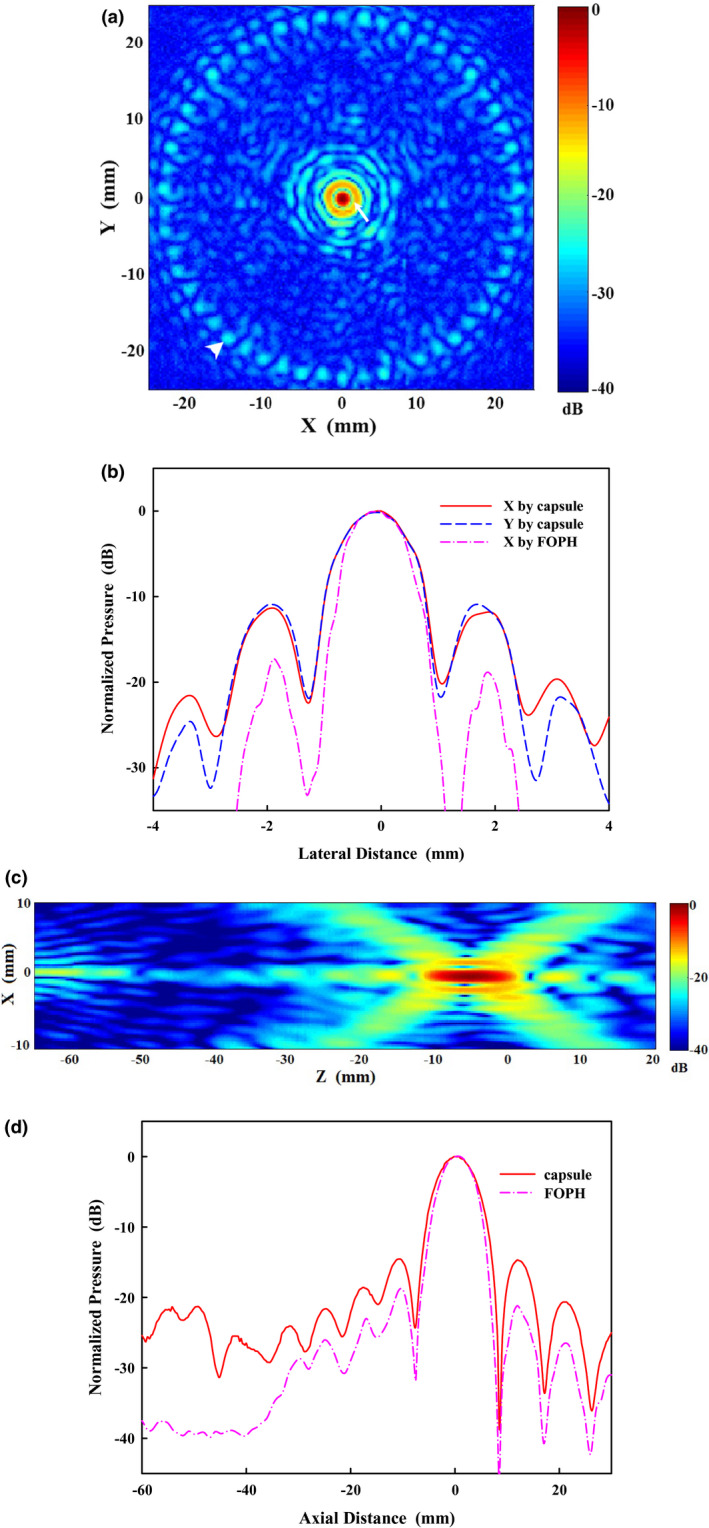
Normalized pressure distribution in the (a) *x‐y* plane, (b) lateral direction, (c) *x‐z* plane, and (d) axial direction. Capsule hydrophone and fiber optic probe hydrophone were used in the field mapping at low and high power output, respectively. White arrow and arrowhead show the first side lobe and grating lobe in the focal plane, respectively.

The acoustic fields of the HIFU transducer in the focal (*x‐y*) and axial (*x‐z*) plane were measured by the capsule hydrophone at the low power (Fig. 3). It is demonstrated that HIFU energy concentrated in a small region with −6 dB beam size of 1.4 × 9.6 mm (W × L). The main lobe had a centralized region of high intensity, 2.5 mm in diameter, and was circumscribed by several side lobes. The locations of the first and second side lobe were 2 and 3.3 mm away from the focus with the amplitudes of −12 and −20 dB, respectively. Grating lobes, consisting of multiple isolated speckles, were found approximately 25 mm away from the focus with the amplitude of −25 dB, which may be associated with the well‐organized, nearly symmetric array configuration of the elements. The acoustic wave converging and diverging along the transducer axis could be observed. The length of the main lobe was about 16.3 mm with the first pre‐ and post‐focal nodes at −7.8 and 8.5 mm, respectively. The first pre‐ and post‐focal side lobes were at −10.5 and 12 mm with the amplitudes of −14.7 and −14.6 dB, respectively. There was an axial grating lobe with the amplitude of < −20 dB at *z* = −45 ~ −60 mm, where is usually at the interface of the tissue and water cushion or inside the patient’s body so that no skin burns or undesired thermal accumulation would be expected if the acoustic coupling is satisfactory.^26^ It was noticed that pressure distribution measured with the FOPH at high power had the same locations of side‐lobe peaks and nodes, but the −6 dB focal size decreased to 1.2 × 9.1 mm and the smaller side lobes (i.e., the first lateral side‐lobe decreased from −11.5 dB at 500 W to −18.1 dB) at an electrical power of 1000 W, which is due to the acoustic nonlinearity.^11^

### 3.B Pressure waveform and power spectrum

**Fig. 4:**
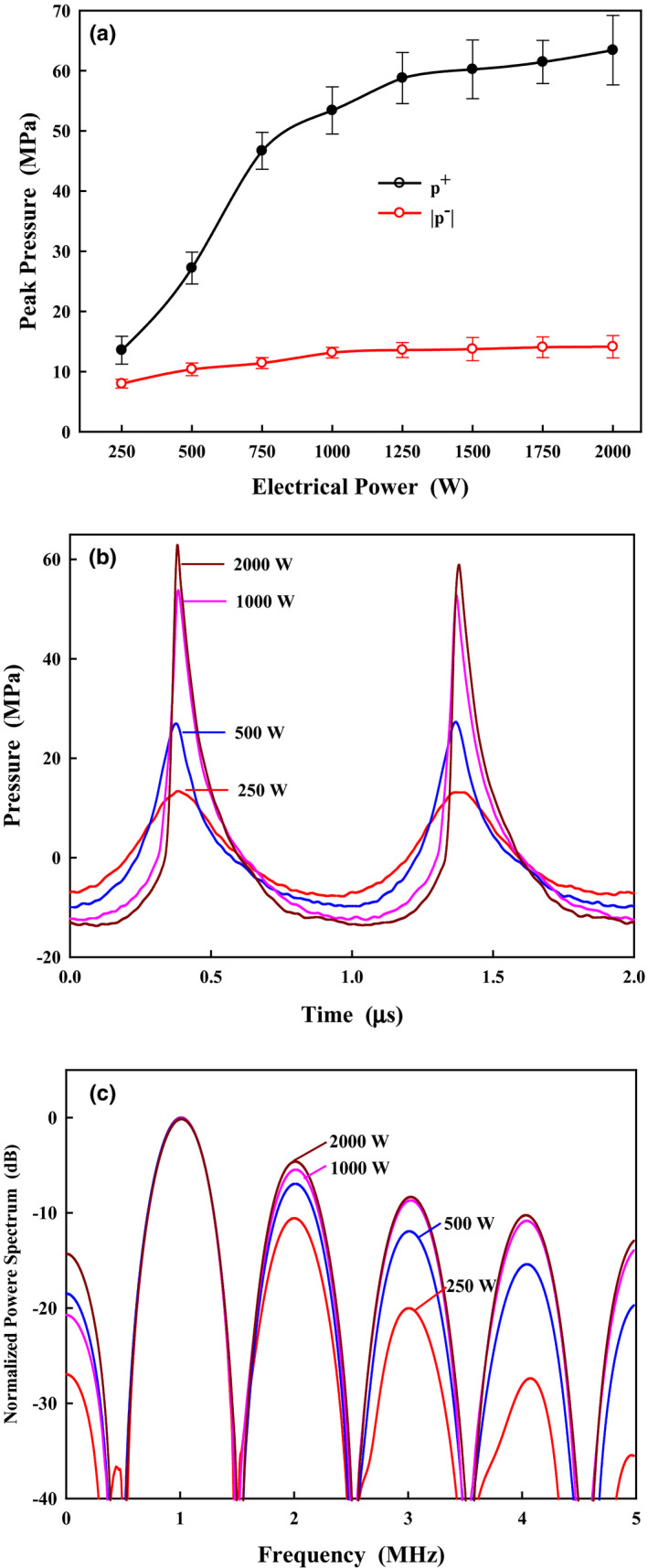
(a) Peak positive and negative pressures measured by fiber optic probe hydrophone at the focus of the lower high‐intensity focused ultrasound transducer with the electrical power up to 2000 W, (b) the measured pressure waveforms, and (c) the corresponding power spectra at the electrical power of 250, 500, 1000, and 2000 W, respectively, normalized by the amplitude of the fundamental frequency.

Peak positive pressure became quickly saturated when the electrical power was larger than 1250 W [Fig. 4(a)]. Representative pressure waveforms at the HIFU focus at the electrical power of 250, 500, 1000, and 2000 W were measured using the FOPH [Fig. 4(b)], and their corresponding power spectra are shown in Fig. 4(c). Increasing the electrical power produced significant nonlinear effects (i.e., the formation of the sharp shock front, the asymmetry between compressional and tensile waves, and the production of more harmonic components). The second harmonic increased from −10.5 dB at 250 W to −4.4 dB at 2000 W, which shows that nonlinearity is a fundamental characteristic of the HIFU field.

**Fig. 5:**
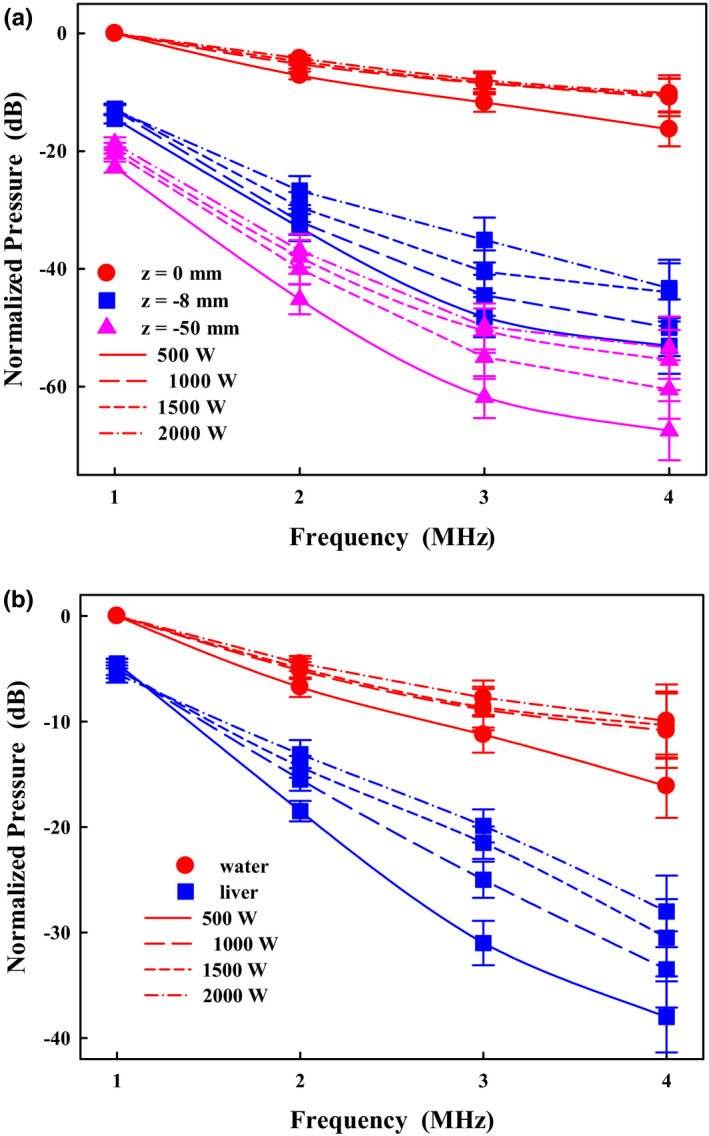
Comparison of the normalized harmonics (a) in the free field at the locations of *z* = 0 (focus), *z* = −8, and *z* = −50 mm, and (b) at *z* = 0 mm with a 6 cm thick bovine liver positioned proximal to the transducer at the electrical power of 500, 1000, 1500, and 2000 W, respectively. Power spectra were normalized by the amplitude of the fundamental frequency at the high‐intensity focused ultrasound focus in the free field.

The generation of harmonics is found to occur usually in the main lobe [Fig. 5(a)]. The amplitudes of the first 3 harmonics at *z* = 0 increased from −7.2 ± 0.7, −11.7 ± 1.6, and −16.3 ± 2.9 dB at 500 W to −4.2 ± 0.5, −8 ± 1.5, and −10.2 ± 3.1 dB at 2000 W, respectively. In comparison at *z* = −8 mm (the peak of the first prefocal side‐lobe), the fundamental and first 3 harmonics were −14.5 ± 0.8, −33 ± 2.3, −48.2 ± 3.4, and −53.1 ± 4.7 dB at 500 W, and −13 ± 0.9, −26.7 ± 2.5, −35.1 ± 3.8, and −43.3 ± 4.8 dB at 2000 W, respectively. The corresponding values of the grating lobe (*z* = −50 mm) were −22.7 ± 0.9, −45.2 ± 2.5, −61.8 ± 3.6, and −67.5 ± 5 dB at 500 W, and −18.7 ± 1.1, −36.9 ± 2.8, −49.8 ± 3.9, and −53.4 ± 5.3 dB at 2000 W, respectively. Overall, there were at least 10 dB differences between the acoustic harmonics at the focus and the prefocal side/grating lobe. Therefore, it suggests that the thermal accumulation away from the focal region is insignificant. Attenuation of the freshly excised bovine liver was measured to be 0.6 ± 0.15 dB/MHz/cm, which is close to the reported values.^27^ After propagating through the tissue sample, the focus was shifted by about 5 mm toward the transducer, and the amplitude of harmonics decreased [Fig. 5(b)]. For example, the differences between the fundamental and first 3 harmonics without and with the intervening sample were 4.5, 11.8, 19.6, and 21.9 dB at 500 W, respectively. Thus, acoustic nonlinearity *ex vivo* and *in vivo* may not have a great contribution to the consequent thermal effect.

### 3.C Acoustic power

**Fig. 6:**
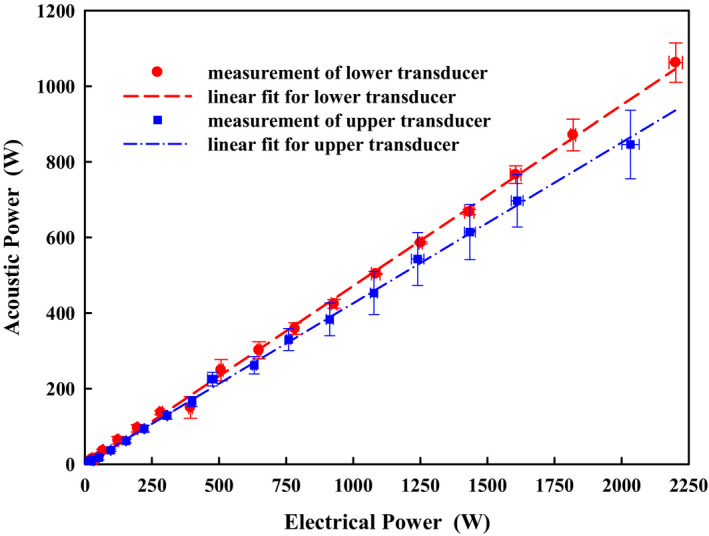
Linear relationship between the electrical power to the upper and lower high‐intensity focused ultrasound transducers measured by a high voltage probe and the acoustic power output measured by the radiation force balance system with the electrical‐to‐acoustic energy conversion efficiencies of 47.8% and 42.5%, respectively.

There was a linear relationship (*R^2^ *= 0.99) between the electrical and acoustic power for both the upper and lower transducers with the electrical‐to‐acoustic energy conversion efficiencies of 42.5% and 47.8%, respectively (Fig. 6). Electrical power to the HIFU transducer was stable with a variation of only 4%. In comparison, the variation in the corresponding acoustic power was a little higher. At the low‐power level (electrical power <100 W) the variation in measured acoustic power was up to 45%, which is due to the low signal‐to‐noise ratio of the load cell for small signals. However, with the increase of electrical power, the variation decreased (~10% and 5% for the upper and lower transducer, respectively). Although the upper and lower HIFU transducers were manufactured identically, the upper HIFU transducer has a little lower electric‐to‐acoustic conversion ratio and higher variation of acoustic power output, which is due to the use of a silicon rubber cushion as the degassed water reservoir instead of a large water cavity as used in the lower transducer. Although the silicon rubber is thin (~0.4 mm) and no significant effect on the pressure waveforms at the focal point was found, the vibration of the silicon cushion with a response to the HIFU pulse‐generated radiation force may dissipate some acoustic energy and introduce additional noise.

### 3.D Single lesion dosimetry

**Fig. 7:**
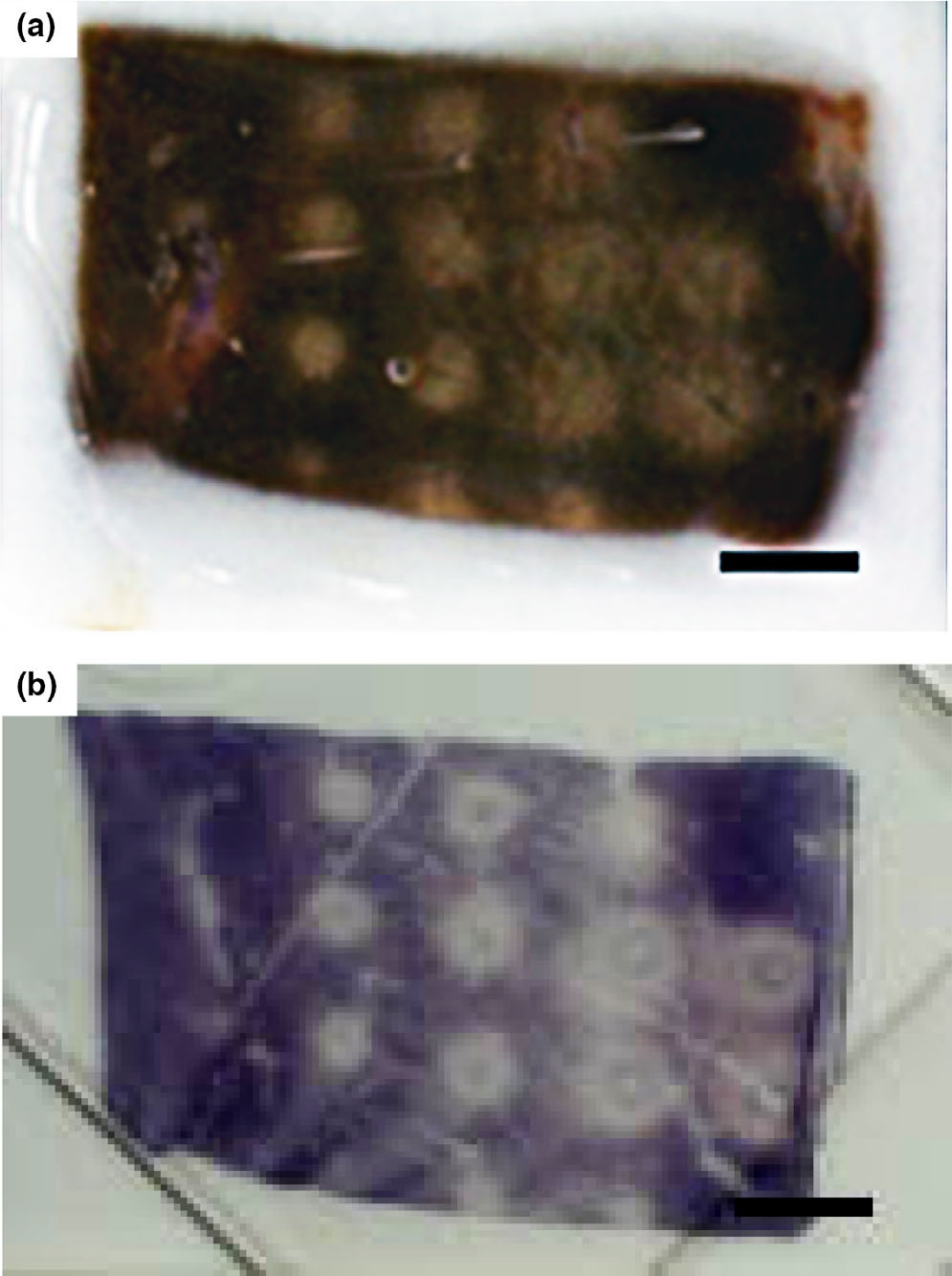
(a) Gross and (b) NADH‐d staining on high‐intensity focused ultrasound‐treated tissue with *in‐situ* acoustic energy of 750 J. In the NADH‐d stained tissue, purple indicates viable tissue and unstained area indicates dead tissue. Scale bar is 5 mm.

**Fig. 8:**
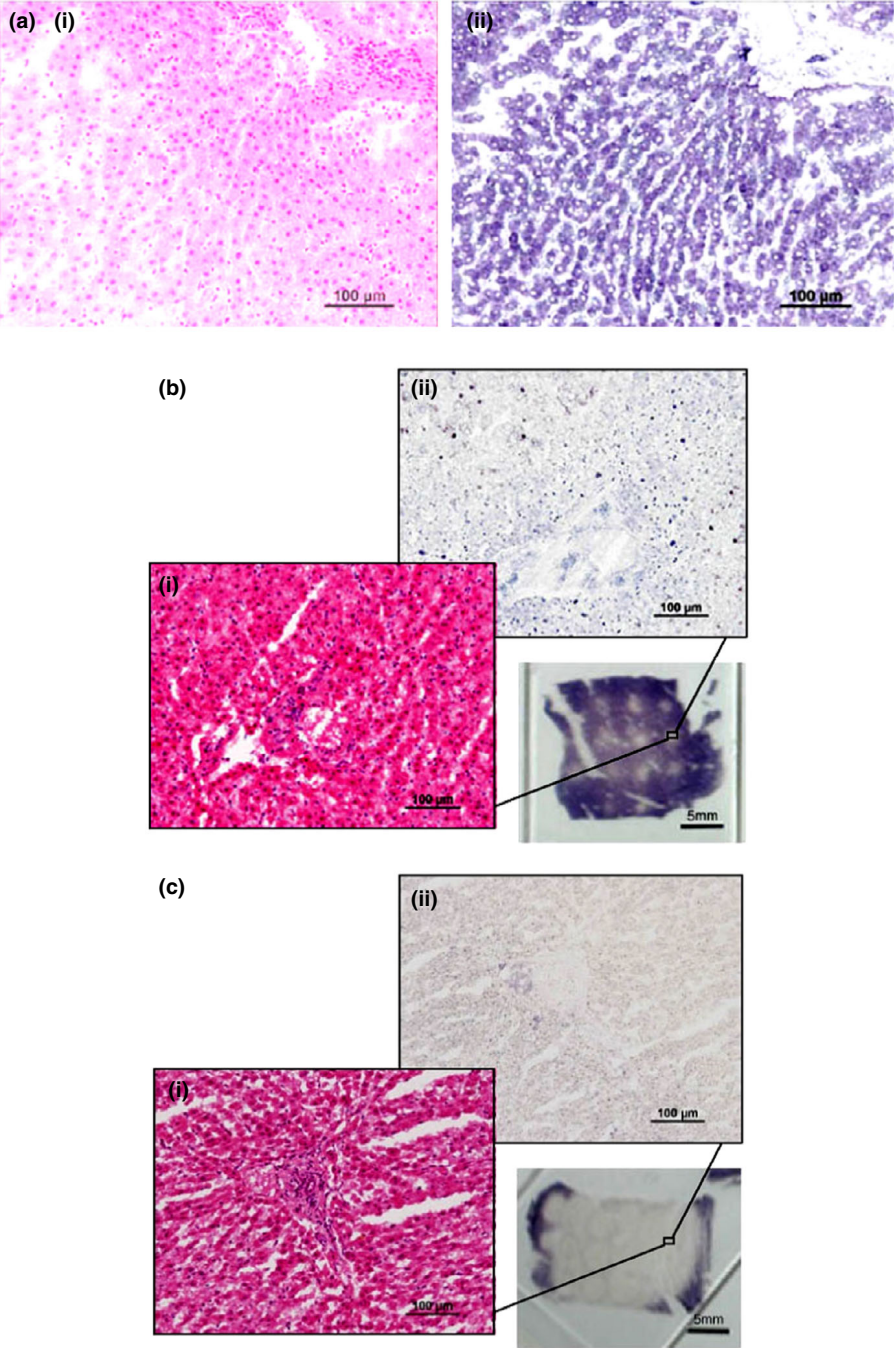
Histology of (a) normal bovine liver and the lesions generated by high‐intensity focused ultrasound with *in‐situ* acoustic energy of (b) 500 J and (c) 1000 J. Tissue is stained with (i) H&E or (ii) NADH‐d. Purple in NADH‐d staining indicates viable tissue while no staining indicates non‐viable cells.

Dosimetry studies in *ex vivo* bovine liver yielded consistent results in 3‐month experiments (*n *> 15) despite some variations due to unavoidable inhomogeneities of the tissue, such as the existence of small vessels. Viability (NADH‐d) staining revealed distinct lesions that corresponded well to areas of discoloration/blanching in the tissue (Fig. 7). However, a blue‐black ring was frequently observed, which is due to an extracellular deposition and an artifact of the staining by examination with higher magnification. Furthermore, lesions generated with low (750 J) and high (1000 J) acoustic energies were evaluated histologically (Fig. 8). In the H&E stained sections, the location of the lesion and cell viability were not apparent as NADH‐d staining. At both acoustic energies, there was no apparent evidence of cell lysis (i.e., hepatocytes) or mechanical disruption of the tissue structure by cavitation or boiling. Hepatic plates in the treated tissue were intact, and there was little disruption of the cellular pattern, but wider sinusoids. Altogether, HIFU‐induced cell damage at the power of no more than 500 W is mainly due to heat fixation rather than mechanical effects.

**Fig. 9:**
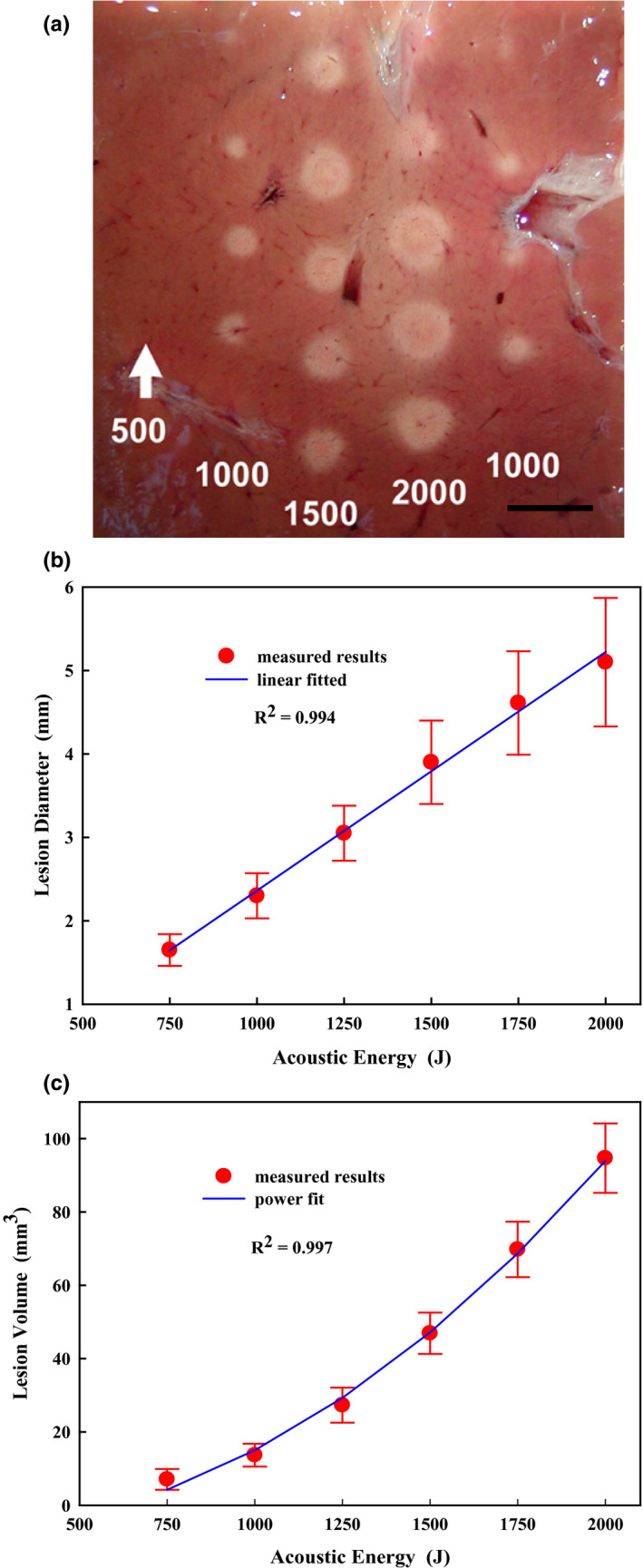
(a) Representative single lesions generated in *ex vivo* bovine liver by using *in‐situ* acoustic energy as shown under each column in Joules. Scale bar represents 7 mm. Dose‐dependence of (b) the maximum lesion size and (c) the lesion volume generated in the *ex vivo* bovine liver by high‐intensity focused ultrasound ablation.

Using the blanched region as an indicator for thermal cell damage, the sizes of HIFU‐generated lesions (*n *> 60) were calculated (Fig. 9). No visible lesion was found in *ex vivo* bovine liver with *in situ* acoustic energy of 500 J, the threshold of HIFU lesion production. It is found that there was a linear relationship between *in situ* acoustic energy and the lesion diameter, increasing from 1.65 ± 0.19 mm at 750 J to 5.1 ± 0.77 mm at 2000 J [Fig. 9(b)]. In addition, the lesion volume was related to *in situ* acoustic energy in a power‐law trend, increasing from 7.1 ± 2.8 mm^3^ at 750 J to 94.7 ± 9.5 mm^3^ at 2000 J with the fitted scaling exponent of 2.12 [Fig. 9(c)].

### 3.E Lesion interaction

**Fig. 10:**
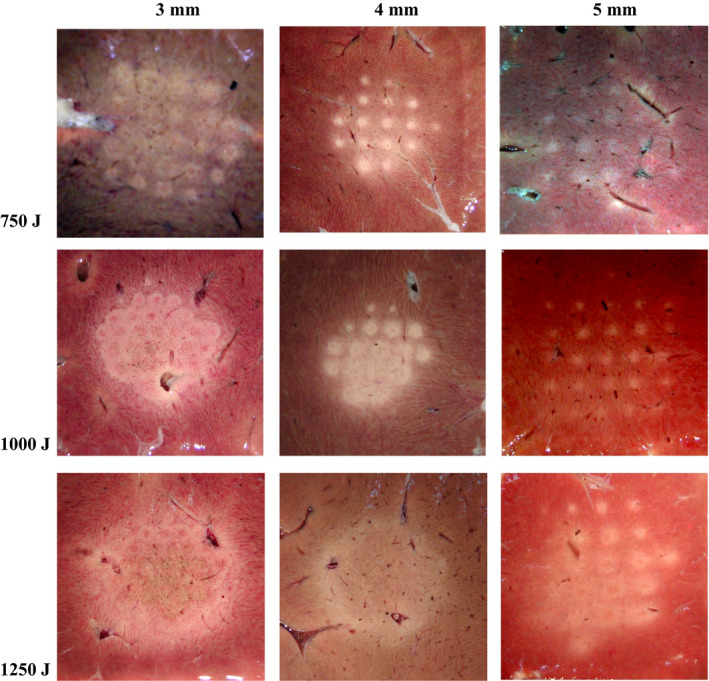
Representative photos of the high‐intensity focused ultrasound‐generated lesions by using different interval spacing between treatment spots and *in‐situ* acoustic energy as shown by the values in each column and row, respectively.

A single layer of lesions with different spacing between spots and *in situ* acoustic energies was generated (Fig. 10). Because of the thermal diffusion effect, the ambient temperature increased with the ongoing of HIFU treatment. As a result, the lesion became progressively larger.^20^ When the lesion size was larger than the interval spacing between nearby spots, the lesion coalescence may occur. Although single lesions were produced in the initial stage with interval spacing of 3 mm (lesion size at 750 and 1000 J were 1.65 ± 0.19 mm and 2.3 ± 0.23 mm, respectively, in Fig. 9(b), a contiguous plane of ablated tissue was found at the end of ablation. With the increment of the interval spacing, the thermal diffusion and lesion coalescence effect became less significant. Almost all individual lesions were produced at *in situ* acoustic energy of 750–1250 J and the interval spacing of 5 mm. Overall, lesion interaction depends on both *in‐situ* acoustic energy and the interval spacing between spots.

### 3.F Temperature elevation

**Fig. 11:**
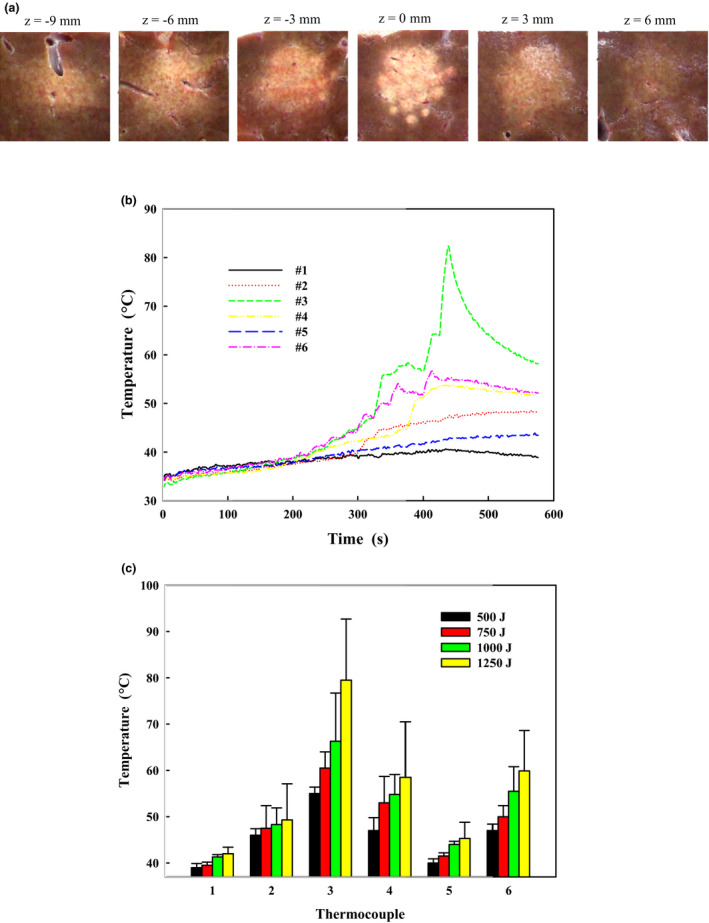
(a) Representative lesions produced by the high‐intensity focused ultrasound ablation with *in‐situ* acoustic energy of 1000 J for each treatment spot, (b) the temperature profiles measured by the thermocouple array, and (c) the relationship of temperature elevations with *in‐situ* acoustic energy from 500 to 1250 J. The location of thermocouples in the treatment target is shown in Fig. 2.

Since the major mechanism of HIFU‐induced cell damage is heat fixation, temperature measurement by the thermocouple array would provide direct evidence of the treatment safety. Representative lesions produced by the extracorporeal HIFU system with *in situ* acoustic energy of 1000 J for each spot are shown in Fig. 11. The initial temperatures were 33–35°C and the maximum temperatures measured by the six thermocouples were 40.6, 48.6, 82.4, 53.8, 43.9, and 56.7°C, respectively. Although the first five thermocouples were positioned symmetrically with respect to the HIFU focus, temperature elevations of the fourth and fifth thermocouple were slightly higher than the first two. The thermal diffusion from the nearby spots with varying amplitude and arrival time (depending on the traveling distance) leads to the multiple‐peak structure in the temperature profile, which is different from the rapid rise and drop due to the cavitation activity.^28^ The corresponding thermal doses were 0.07, 86.7, 5.3 × 10^10^, 2781.4, 3.0, and 8553.6 CEM, respectively. With the increase of *in‐situ* acoustic energy, the temperature in the liver sample increased correspondingly. The maximum temperatures at the HIFU focus were 55 ± 1.4°C at 500 J, 60.5 ± 3.5°C at 750 J, 66.3 ± 10.4°C at 1000 J, and 79.5 ± 13.2°C at 1250 J, respectively. Less than 10°C temperature elevation (maximum temperature increasing from 39 ± 0.9°C to 42.1 ± 1.4°C, and from 40 ± 0.9°C to 45.3 ± 3.5°C for the first and fifth thermal couple, respectively, at *in situ* acoustic energy of 500–1250 J) at 1 cm away from the treatment target,^29^ which is the minimum distance between the boundary of pancreatic cancer and nearby tissue or organ (i.e., vessel and nerve) as required by FDA, suggests that HIFU energy and its induced thermal effect is concentrated in a well‐defined region with little side effects to the surroundings.

## 4 DISCUSSIONS

Extracorporeal HIFU devices are used for targets lying within the breast, abdomen, brain or limbs. Transcutaneous abdominal treatments require a suitable acoustic window, providing an unobstructed propagation path (i.e., no intervening bowel gas) and satisfactory coupling at the wave entry site (skin) for the focused beam. HIFU treatments are guided using either ultrasound (i.e., FEP‐BY02) or MRI imaging (i.e., ExAblate 2000 of InSightec, Israel, and Sonalleve of Profound Medical, Canada). MRI has excellent anatomical resolution and high sensitivity for tumor detection. Using MR thermometry, it enables the calculation of thermal dose to achieve cytotoxicity and, subsequently, closed‐loop control of energy deposition with a temperature accuracy of 1°C, the spatial resolution of 1 mm, and temporal resolution of 1 s during HIFU ablation. Although MRI is superior to sonography in obese patients,^30^ it is expensive, labor‐intensive, and of lower spatial resolution in some cases. In comparison, sonography, whose transducer is usually incorporated into the treatment head, allows real‐time imaging of the ablation process at a much lower cost. Sonography guidance uses the same form of energy as HIFU therapy to exam the condition of the acoustic window. Therefore, if the target cannot be well visualized, HIFU therapy will become ineffective, and unintentionally thermal injury may be produced. According to our preclinical *in vivo* experience using this FEP‐BY02 system, sustained thermal injury to the abdominal wall occurred only if the image quality was either “fair” or “poor.”^19^ Abdominal wall injury was not seen in any treatments where the image quality was rated as “good.” In addition, the thermally ablated region is not visible on the standard B‐mode sonography unless gas bubbles have been induced. Although radiation force imaging technique has been demonstrated as an effective approach to detecting HIFU‐induced lesion,^31^ application in real (or quasi‐real) time has not been implemented in practice. Furthermore, ultrasonic thermography techniques, such as analyzing the backscattered ultrasound signal,^32^, ^33^ with the detectable temperature up to more than 65°C *in vivo* are under development. Overall, the lack of lesion and temperature monitoring functions limited the wide acceptance of sonography‐guided HIFU therapy.

The experiments of acoustic field characterization and lesion evaluation were repeated many times within 3 months. Small variations (<5%) of measurement from different operators using the same protocols suggest the reliability of the HIFU system and our calibration method. The measured pressure waveform and distribution of the HIFU field had a good agreement with our numerical simulation based on the wide‐angle parabolic equation (data not included). Fiber optic hydrophone has the advantages of the small sensing element, broad bandwidth, robustness to cavitation damage, immunity to electromagnetic interference, and easy preparation of a new tip. Thus, it has been used widely in measuring the spatial distribution of the peak particle velocity within the focus of a HIFU transducer,^34^, ^35^ acoustic pressure and temperature simultaneously with a polymer film Fabry‐Pérot interferometer deposited at the fiber tip,^36^ and cavitation activities.^37^ Because of the large size of the HIFU transducers and limited maximum acoustic power measurement in the commercial radiation force balance (i.e., 30 W for the ultrasound power meter from Ohmic Instruments and 100 W for RFB‐2000 from Onda) a lab‐built one was used in this study. It showed good reliability and repeatability despite low signal‐to‐noise ratio at low power output (<100 W). The calibration of the HIFU field was done in water for its output and comparison with the other systems. However, the measured characteristics cannot be transferred to the *in vivo* environment directly because of the heterogeneity of tissue, challenges to determine its acoustic properties *in situ*, and the defocusing effect of the acoustic beam through the tissue. Patient‐specific planning is possible by generating layered surface model using 2D/3D segmentation on pre‐therapy images (i.e., CT or MRI), simulating the beam distribution and temperature elevation, and visualizing the integrated 3D anatomy and HIFU beam simulation.^38^

Characterization of medical ultrasound device using existing IEC standards is generally the description of the acoustic field and the generated thermal field, but not related to the effectiveness or clinical performance directly. For example, beam width is normally determined from the positions where the temporal average pressure falls to 6 dB below the peak value in the focal plane. In shock wave lithotripter, the peak positive pressure at the focus varies from 40 to 120 MPa, depending on the output voltage, focusing gain, and the methods of shock wave generation. As a result, at the edge of beam width the pressure could be up to 60 MPa, which is much higher than the stone fragmentation threshold (~10 MPa). Therefore, the −6 dB beam width simply defines the quality of focusing, but does not illustrate the energy level and quantify the fragmentation ability of a lithotripter. The 10 MPa fragmentation zone introduced by the German Society of Shock Wave Lithotripsy is appropriate for realistic comparisons of lithotripter performance.^39^, ^40^ Similarly, measurement of energy deposition in the focal region and the consequent thermal dose for protein denaturation and irreversible lesion production may be more suitable for evaluating the ablation ability of HIFU devices. In addition, the characteristics of nonlinear acoustic at the high‐power level, such as the distortion in the pressure waveform profile, the generation of harmonics in the spectrum, and the corresponding mechanical effects (i.e., bubble cavitation), would discern itself from diagnostic or physiotherapy fields.

In the acoustic field, ultrasound‐induced cell damage may be due to three mechanisms: heat fixation, cavitation, and boiling. Cell lysis is characterized by structural damage at the medically relevant frequencies and intensities and closely correlates with the occurrence of acoustic cavitation at the cell membrane. However, the surviving intact cells are largely unaffected with respect to growth rate and progression through the cell cycle.^41^ Biological tissue becomes thermally coagulated with consequent discoloration and blanching of the tissue, formation of derivatives of collagen (i.e., glucose), and contraction of collagen if its temperature exceeds 60–65°C.^42^ If the temperature of tissue reaches the boiling of intra‐ or extracellular liquid (~100°C), rapid vaporization will occur. HIFU‐induced boiling would cause contraction and shrinkage of tissue, known as desiccation effect. In addition, the shock wave generated from the explosive boiling can subsequently cause damage to cells relatively far away from the point of impact. A combination of bubble cavitation and boiling is presumed to be the major cause of the significant grey‐scale changes in real‐time B‐mode sonography.^28^ However, this hyperecho is naturally transient and does not correlate well with the tissue response in the nonrandomized clinical trials.^43^, ^44^ It is interesting to note that sometimes the center of HIFU‐ablated cancer in H&E staining looked similar to viable cells, maintaining their characteristics of cytologic staining and nuclear chromatin without any signs of breakdown. However, electron microscopy revealed that the cytoplasm of those normal‐appearing cancer cells contained vacuoles, cell membranes were disintegrated, and organelle structures were not identified, suggesting an irreversible cell death and the preservation of cellular structure induced by thermal fixation, instead of incomplete coagulation necrosis.^44^ As a result, the central part of the ablated tumor resisted degradation because the wound‐healing process could not extend to this region immediately after HIFU treatment. In contrast, in the peripheral region, cancer cells had the typical characteristics of lethal and irreversible cell damage as coagulation necrosis (i.e., pyknotic nuclei, nuclear disruption, and disappearance). Along the margin of the ablation, a narrow cellular band of fibrous tissue could be identified, with the presence of fibroblasts, inflammatory cells, collagen fibrin, and capillary network. Therefore, NADH‐diaphorase stain is more accurate and objective than H&E staining in assessing acute cell death because it is based on the presence or absence of enzyme function instead of changes in the cellular structure. Thermal fixation dominates cell damage in HIFU ablation, which is based on the lack of histologic evidence of cavitation, the linear changes of temperature with power, and no sharp elevation of measured temperatures. In this study and future clinical trials, the electrical power output is only up to 500 W. Heat fixation is assumed as the dominant mechanism so that *in‐situ* acoustic energy was developed for delivering almost the same absorbed acoustic energy to different patients. Although there are variations of acoustic attenuation in the same type of tissue, such protocol will allow us a more reasonable comparison of HIFU outcome rather than using only the operation parameters (i.e., power and effective exposure time).

## 5 CONCLUSION

A clinical extracorporeal HIFU system was characterized using our protocols for the acoustic field (i.e., acoustic pressure waveform, pressure distribution, and acoustic power) up to electrical power of 2000 W. Comparison of acoustic pressure waveforms and their corresponding spectra at the focus in the free field with those ones after propagating through tissue samples demonstrated that the acoustic nonlinearity is less *ex vivo*. An *in situ* acoustic energy in HIFU ablation was established and then tested in *ex vivo* experiments at the power output up to 500 W. Linear relationship was found between the HIFU‐induced lesion size and *in situ* acoustic energy beyond the threshold of 500 J. Thermocouple array measurement showed that the high‐temperature elevation only occurs at the focal region of HIFU and being 10 mm away from the target boundary would lead to a change of no more than 10°C, which suggests minor side effect to the surrounding healthy tissue. This extracorporeal system has already obtained the FDA’s approval for its clinical trial on the palliation of pain associated with pancreatic cancer in the USA. Our HIFU calibration approaches and *ex vivo* evaluation protocols can also be applied for the other HIFU systems.

## AUTHORS CONTRIBUTIONS

Y.F. Zhou, B.W. Cunitz, and B. Dunmire did the measurement and processed the data. Y. N. Wang did pathological study on the treated tissue samples. S.G. Karl simulated the acoustic field. C.W. Warren organized the investigation. S. Mitchell and J.H. Hwang supervised the project. Y.F. Zhou and J.H. Hwang wrote the manuscript.

## CONFLICT OF INTEREST

The authors conducted all experiments under the requirement of FDA for FEP‐BY02 HIFU system for IDE application (#100169).
